# Supplementary material for: The genetic architecture of protein stability
Source: Nature. 2024 Sep 25;634(8035):995–1003. doi: 10.1038/s41586-024-07966-0 (PMC11499273; doi:10.1038/s41586-024-07966-0)
Supplement: Supplementary file 1 — Reporting Summary [file 41586_2024_7966_MOESM1_ESM.pdf]

nature portfolio

Corresponding author(s): Andre J. Faure, Ben Lehner

Last updated by author(s): Jul 26, 2024

Reporting Summary

Nature Portfolio wishes to improve the reproducibility of the work that we publish. This form provides structure for consistency and transparency in reporting. For further information on Nature Portfolio policies, see our Editorial Policies and the Editorial Policy Checklist.

Statistics

For all statistical analyses, confirm that the following items are present in the figure legend, table legend, main text, or Methods section.

n/a	Confirmed	
☐	☒	The exact sample size (*n*) for each experimental group/condition, given as a discrete number and unit of measurement
☐	☒	A statement on whether measurements were taken from distinct samples or whether the same sample was measured repeatedly
☐	☒	The statistical test(s) used AND whether they are one- or two-sided<br>*Only common tests should be described solely by name; describe more complex techniques in the Methods section.*
☒	☐	A description of all covariates tested
☐	☒	A description of any assumptions or corrections, such as tests of normality and adjustment for multiple comparisons
☐	☒	A full description of the statistical parameters including central tendency (e.g. means) or other basic estimates (e.g. regression coefficient) AND variation (e.g. standard deviation) or associated estimates of uncertainty (e.g. confidence intervals)
☐	☒	For null hypothesis testing, the test statistic (e.g. F, t, r) with confidence intervals, effect sizes, degrees of freedom and P value noted<br>*Give P values as exact values whenever suitable.*
☒	☐	For Bayesian analysis, information on the choice of priors and Markov chain Monte Carlo settings
☒	☐	For hierarchical and complex designs, identification of the appropriate level for tests and full reporting of outcomes
☐	☒	Estimates of effect sizes (e.g. Cohen's *d*, Pearson's *r*), indicating how they were calculated

Our web collection on statistics for biologists contains articles on many of the points above.

Software and code

Policy information about availability of computer code

Data collection	FastQ files from paired-end sequencing of all bindingPCA and abundancePCA experiments were processed with DiMSum v1.3 using default settings with minor adjustments: https://github.com/lehner-lab/DiMSum. All experimental design files and bash scripts with command-line options required for running DiMSum on these datasets are available at https://github.com/lehner-lab/archstabms.
Data analysis	Source code for fitting thermodynamic models (MoCHI v0.9) is available at https://github.com/lehner-lab/MoCHI. Source code for all downstream analyses, including DiMSum and MoCHI configuration files and to reproduce all figures described here is available at https://github.com/lehner-lab/archstabms. An archive of this repository is also publicly available on Zenodo at https://zenodo.org/doi/10.5281/zenodo.11671164. Chemical bonds or interactions were calculated using the GetContacts software package (https://github.com/getcontacts/getcontacts) with version status: July 25th 2023. PyMOL v2.5.2 was used to fill missing hydrogens. FoldX v4 was used to restore the wild-type Proline at position 54 that is mutated in the reference crystal structure (PDB: 2VWF, “PositionScan” command).

For manuscripts utilizing custom algorithms or software that are central to the research but not yet described in published literature, software must be made available to editors and reviewers. We strongly encourage code deposition in a community repository (e.g. GitHub). See the Nature Portfolio guidelines for submitting code & software for further information.

Data

Policy information about availability of data

All manuscripts must include a data availability statement. This statement should provide the following information, where applicable:

- Accession codes, unique identifiers, or web links for publicly available datasets
- A description of any restrictions on data availability
- For clinical datasets or third party data, please ensure that the statement adheres to our policy

All DNA sequencing data have been deposited in the Gene Expression Omnibus (GEO) with accession number GSE246322: https://www.ncbi.nlm.nih.gov/geo/query/acc.cgi?acc=GSE246322. Associated fitness measurements and free energies are provided in Supplementary tables 4 and 5. Shallow double mutant ddPCA DNA sequencing data for GRB2-SH3 and PSD95-PDZ3 is available in GEO with accession number GSE184042: https://www.ncbi.nlm.nih.gov/geo/query/acc.cgi?acc=GSE184042 and the processed data used in this study can be found in Supplementary Tables 6 and 7 of the corresponding publication (DOI: 10.1038/s41586-022-04586-4). Protein structures for GRB2-SH3 (Entry ID: 2VWF) and SRC (Entry ID: 2SRC) are available from the Protein Data Bank (PDB): https://www.rcsb.org/

Research involving human participants, their data, or biological material

Policy information about studies with human participants or human data. See also policy information about sex, gender (identity/presentation), and sexual orientation and race, ethnicity and racism.

Reporting on sex and gender	N/A
Reporting on race, ethnicity, or other socially relevant groupings	N/A
Population characteristics	N/A
Recruitment	N/A
Ethics oversight	N/A

Note that full information on the approval of the study protocol must also be provided in the manuscript.

Field-specific reporting

Please select the one below that is the best fit for your research. If you are not sure, read the appropriate sections before making your selection.

☒ Life sciences ☐ Behavioural & social sciences ☐ Ecological, evolutionary & environmental sciences

For a reference copy of the document with all sections, see nature.com/documents/nr-reporting-summary-flat.pdf

Life sciences study design

All studies must disclose on these points even when the disclosure is negative.

Sample size	Sample sizes during the construction of the mutant libraries and the yeast competition experiments were always several fold larger than the bottlenecked library size to ensure losing as few amino acid variants as possible during the experiments. The minimum number of yeast transformants in each of the bulk competition replicates (the strongest bottleneck in the experimental design) was calculated so that mutations in the library would be found on average in 20-25 different cells.
Data exclusions	Sequencing reads that did not pass the QC filters using DiMSum v1.3 (https://github.com/lehner-lab/DiMSum) were excluded. For mutagenesis library 1 read counts for all variants were adjusted by subtracting the expected number of sequencing errors derived from the wild-type-only sample and proportional to the total sequencing library size of each sample.
Replication	All bulk yeast competitions per assay and protein library were performed in triplicates. All attempts of replication were successful.
Randomization	Not relevant for this study because mutant libraries were created systematically and screened in bulk selections experiments.
Blinding	Not relevant for this study because mutant libraries were created systematically and screened in bulk selections experiments.

Reporting for specific materials, systems and methods

We require information from authors about some types of materials, experimental systems and methods used in many studies. Here, indicate whether each material, system or method listed is relevant to your study. If you are not sure if a list item applies to your research, read the appropriate section before selecting a response.

Materials & experimental systems

n/a	Involved in the study
☒	☐ Antibodies
☐	☒ Eukaryotic cell lines
☒	☐ Palaeontology and archaeology
☒	☐ Animals and other organisms
☒	☐ Clinical data
☒	☐ Dual use research of concern
☒	☐ Plants

Methods

n/a	Involved in the study
☒	☐ ChIP-seq
☒	☐ Flow cytometry
☒	☐ MRI-based neuroimaging

Eukaryotic cell lines

Policy information about cell lines and Sex and Gender in Research

Cell line source(s): Saccharomyces cerevisiae BY4742 (MATα his3Δ1 leu2Δ0 lys2Δ0 ura3Δ0)

Authentication: The cell line was not authenticated

Mycoplasma contamination: Not tested for Mycoplasma (not applicable)

Commonly misidentified lines (See ICLAC register): *Name any commonly misidentified cell lines used in the study and provide a rationale for their use.*

Plants

Seed stocks: *Report on the source of all seed stocks or other plant material used. If applicable, state the seed stock centre and catalogue number. If plant specimens were collected from the field, describe the collection location, date and sampling procedures.*

Novel plant genotypes: *Describe the methods by which all novel plant genotypes were produced. This includes those generated by transgenic approaches, gene editing, chemical/radiation-based mutagenesis and hybridization. For transgenic lines, describe the transformation method, the number of independent lines analyzed and the generation upon which experiments were performed. For gene-edited lines, describe the editor used, the endogenous sequence targeted for editing, the targeting guide RNA sequence (if applicable) and how the editor was applied.*

Authentication: *Describe any authentication procedures for each seed stock used or novel genotype generated. Describe any experiments used to assess the effect of a mutation and, where applicable, how potential secondary effects (e.g. second site T-DNA insertions, mosiacism, off-target gene editing) were examined.*
